# Illinois

**Published:** 1897-04

**Authors:** 


*Illinois.* The movement in Illinois to establish a veterinary college at the University of Illinois has taken shape in the form of Senate Bill No. 204, which provides for the creation of such a department on an adequate scale, and has very earnest support throughout the State. If this wise movement is to be accomplished, the aid of every veterinarian in the State will be needed.

The bill to regulate the practice of veterinary science is in a fair way for favorable consideration. If the State is going to create a school of veterinary medicine, it should be prompt and eager to properly protect those who qualify themselves for this work.

